# Adherence to treatment with artemether–lumefantrine or amodiaquine–artesunate for uncomplicated malaria in children in Sierra Leone: a randomized trial

**DOI:** 10.1186/s12936-018-2370-x

**Published:** 2018-06-04

**Authors:** Kristin Banek, Emily L. Webb, Samuel Juana Smith, Daniel Chandramohan, Sarah G. Staedke

**Affiliations:** 1 Department of Clinical Research, London School of Hygiene and Tropical Medicine, Keppel Street, London, WC1E 7HT UK; 2 MRC Tropical Epidemiology Group, London School of Hygiene and Tropical Medicine, Keppel Street, London, WC1E 7HT UK; 3 National Malaria Control Programme, Ministry of Health and Sanitation-Sierra Leone, Freetown, Sierra Leone; 4 Department of Disease Control, London School of Hygiene and Tropical Medicine, Keppel Street, London, WC1E 7HT UK

**Keywords:** Malaria, Artemisinin-based combination therapy (ACT), Adherence, Compliance, Artemether–lumefantrine, Amodiaquine, Artesunate, Fixed-dose combination, Co-formulated

## Abstract

**Background:**

Prompt, effective treatment of confirmed malaria cases with artemisinin-based combination therapy (ACT) is a cornerstone of malaria control. Maximizing adherence to ACT medicines is key to ensuring treatment effectiveness.

**Methods:**

This open-label, randomized trial evaluated caregiver adherence to co-formulated artemether–lumefantrine (AL) and fixed-dose amodiaquine–artesunate (AQAS) in Sierra Leone. Children aged 6–59 months diagnosed with malaria were recruited from two public clinics, randomized to receive AL or AQAS, and visited at home the day after completing treatment. Analyses were stratified by site, due to differences in participant characteristics and outcomes.

**Results:**

Of the 784 randomized children, 680 (85.6%) were included in the final per-protocol analysis (340 AL, 340 AQAS). Definite adherence (self-reported adherence plus empty package) was higher for AL than AQAS at both sites (Site 1: 79.4% AL vs 63.4% AQAS, odds ratio [OR] 2.16, compared to probable adherence plus probable or definite non-adherence, 95% confidence interval [CI] 1.34–3.49; p = 0.001; Site 2: 52.1% AL vs 37.5% AQAS, OR 1.53, 95% CI 1.00–2.33, p = 0.049). However, self-reported adherence (ignoring drug package inspection) was higher for both regimens at both sites and there was no strong evidence of variation by treatment (Site 1: 96.6% AL vs 95.9% AQAS, OR 1.19, 95% CI 0.39–3.63, p = 0.753; Site 2: 91.5% AL vs 96.4% AQAS, OR 0.40, 95% CI 0.15–1.07, p = 0.067). In Site 2, correct treatment (correct dose + timing + duration) was lower for AL than AQAS (75.8% vs 88.1%, OR 0.42, 95% CI 0.23–0.76, p = 0.004). In both sites, more caregivers in the AQAS arm reported adverse events (Site 1: 3.4% AL vs 15.7% AQAS, p < 0.001; Site 2: 15.2% AL vs 24.4% AQAS, p = 0.039).

**Conclusions:**

Self-reported adherence was high for both AL and AQAS, but varied by site. These results suggest that each regimen has potential disadvantages that might affect adherence; AL was less likely to be taken correctly at one site, but was better tolerated than AQAS at both sites. Measuring adherence to anti-malarials remains challenging, but important. Future research should focus on comparative studies of new drug regimens, and improving the methodology of measuring adherence.

*Trial registration*: Clinicaltrials.gov, NCT01967472. Retrospectively registered 18 October 2013, https://clinicaltrials.gov/ct2/show/NCT01967472

**Electronic supplementary material:**

The online version of this article (10.1186/s12936-018-2370-x) contains supplementary material, which is available to authorized users.

## Background

Universal coverage of diagnostic testing and prompt, effective treatment with artemisinin-based combination therapy (ACT) are key malaria control strategies [1, 2]. However, the effectiveness of malaria case management depends on multiple factors, including availability of (and access to) treatment with ACT, prescriber compliance to guidelines, and importantly, patient (or caregiver) adherence to treatment regimens [3, 4]. Strategies to improve adherence, including co-packaging anti-malarial drugs into blister packs [5, 6], and co-formulating the partner drugs of ACT into a single tablet, have been applied successfully [7, 8]. Although co-formulation may improve the accuracy of prescription and ease of administration of ACT [9], it may not solve all treatment challenges, including complex dosing, bitter taste, and side effects [10–13]. Furthermore, evidence of the impact of co-formulation on adherence to ACT remains limited [3].

Malaria remains a major health problem in Sierra Leone, exacerbated by the Ebola outbreak in 2014, which overwhelmed an already fragile health system [14–18]. In 2004, co-packaged amodiaquine plus artesunate (AQ + AS) was adopted as the first-line recommended treatment of malaria, primarily due to affordability and availability, with artemether–lumefantrine (AL) as an alternative if AQ + AS was not available, or was ineffective [19]. In 2008, a study of adherence to co-packaged AQ + AS in Sierra Leone concluded that only 48.7% of participants were probably or definitely adherent to treatment [20]. In 2013, with support from The Global Fund to Fight AIDS, Tuberculosis and Malaria (GFATM) the fixed-dose combination version of amodiaquine–artesunate (AQAS) replaced the co-packaged AS + AQ regimen, with AL remaining as an alternate. However, in 2015, following the mass drug administration (MDA) campaign during the Ebola outbreak, AL replaced AQAS as the treatment of choice for uncomplicated malaria in Sierra Leone, with AQAS now as the alternate [21]. The impact of these changes in anti-malarial drug policy on patient adherence and the overall effectiveness of malaria treatment in Sierra Leone remains unclear. To date, no studies have evaluated adherence to either AL or AQAS in Sierra Leone.

Only three studies have compared the adherence to multiple ACT regimens in Africa; however, the primary outcome of all three studies was treatment effectiveness; adherence was evaluated as a secondary outcome [22–24]. Only one of these studies, conducted in Benin, compared AL and AQAS [24], finding no significant difference in full adherence to AL compared to AQAS (83.0% vs 91.0%; p = 0.16). To address this gap in evidence, an open-label, randomized trial was conducted in Sierra Leone to compare caregiver adherence to co-formulated AL to that of AQAS for treatment of uncomplicated malaria in children aged 6–59 months.

## Methods

### Study sites

**Fig. 1:**
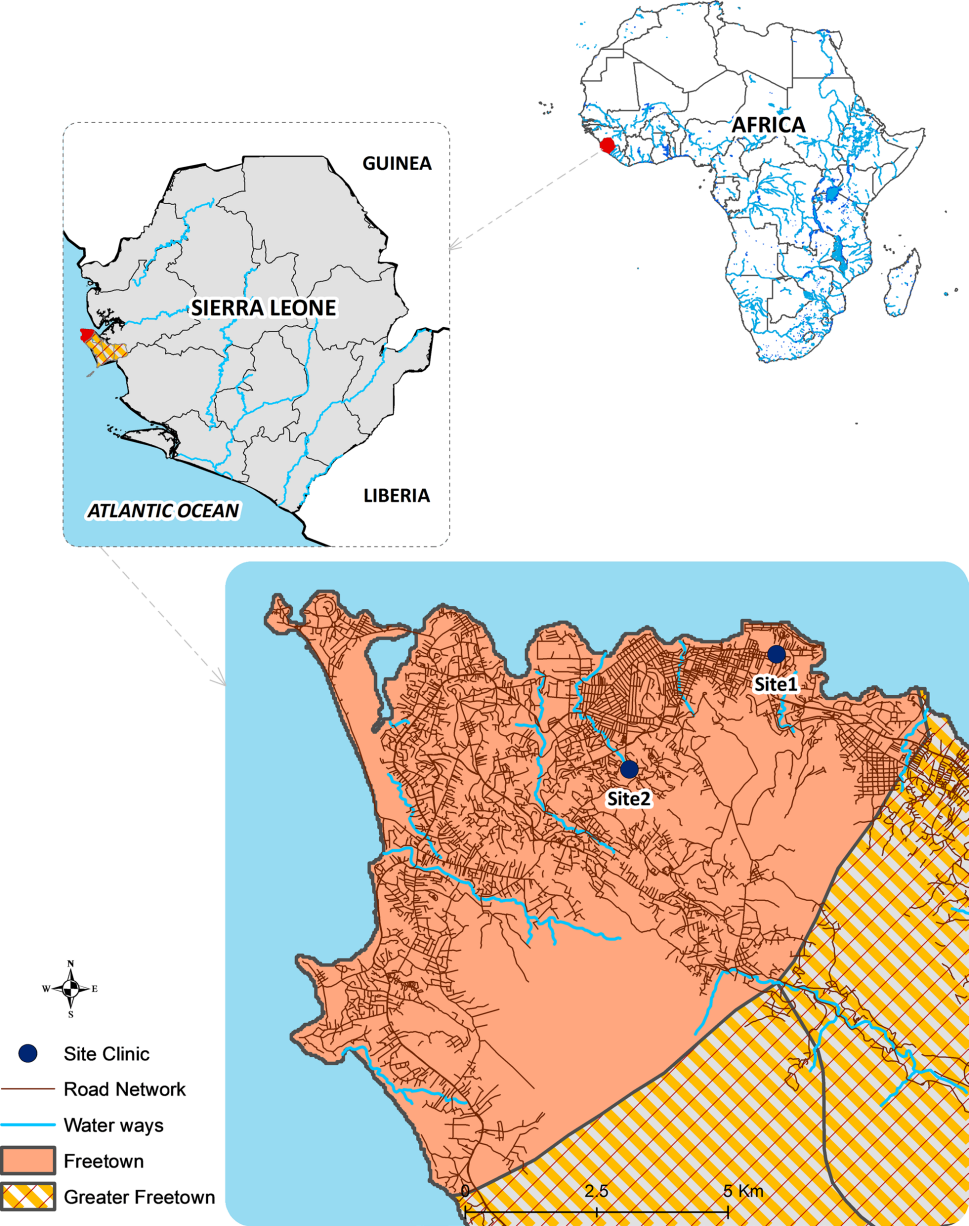
Map of the study area. Blue dots—study sites

The study was conducted at two government-run outpatient facilities in Freetown, Sierra Leone (Fig. 1). Both sites were chosen for their high patient loads, similar catchment population and patient numbers as well as the size of staff (10–15 health workers per site). Site 1 is located in a densely populated area in the eastern part of Freetown. The clinic has an estimated catchment population of 21,324 people and manages approximately 1000 patients per month, half of whom are children under 5 years of age presenting with fever. In 2012, the clinic had 400 children under five with confirmed malaria every month (extracted from routine health data). Site 2 is located in the western part of Freetown. This clinic has an estimated catchment population of 27,855 people, with approximately 800 patient visits per month. In 2012, 60% of patients were children under 5 years of age presenting with fever, including an average of 240 confirmed malaria cases each month (routine health data).

### Study procedures

Children were enrolled in the study, if they met the following inclusion criteria: (1) age 6–59 months; (2) complaint of fever or history of fever; (3) living within 5 km of health facility; (4) no evidence of severe malaria or danger signs (i.e. inability to eat/drink, extreme lethargy, inability to sit/stand, difficulty breathing, jaundice, severe dehydration, convulsion or persistent vomiting) [2, 21, 25]; (5) not referred to another health facility; (6) not previously enrolled in the study; and (7) written informed consent to participate in the study provided by their parent or guardian. During the consenting process, caregivers were informed about the purpose of the study, but were not told that they would be visited at home or that their adherence to treatment guidelines would be assessed.

Following enrolment, study participants were subject to the standard of care provided at the health centres. All participants first underwent testing for malaria using rapid diagnostic tests (RDTs) at the health centre laboratory, and then saw a consulting health worker for a clinical evaluation. Study staff recorded RDT results onto case record forms, and observed patient-provider consultations for all participants. Children who had a positive RDT and were diagnosed with malaria by the health worker were randomly assigned to receive treatment with either AL or AQAS and were scheduled for a home visit on Day 4. If the RDT was negative for malaria, the child was excluded from the study and was provided standard care by the health worker.

### Study medication

Co-formulated AL (Coartem Dispersible^®^: Novartis) was procured by the research team and provided to both sites for the purpose of this study. Fixed-dose AQAS (Winthrop^®^: Sanofi-Aventis) was available at both study sites through the standard government supply chain system. All study medications were produced by manufacturers pre-qualified by the World Health Organization (WHO) and approved by the Pharmacy Board of Sierra Leone, and were prescribed according to standard national and WHO treatment guidelines [25, 26].

Participants randomized to treatment with AL received 20/120 mg tablets dosed appropriately twice a day for 3 days. Health workers prescribed treatment based on weight when possible; if weighing scales were not available, treatment was prescribed by age. Children aged 6–11 months (or weighing 5–15 kg) received 1 tablet per dose (infant dose), and those aged 12–59 months of age (or weighing > 15 to 20 kg) received 2 tablets per dose (child dose). Participants randomized to treatment with AQAS received one tablet dosed appropriately for age (or weight) once daily for 3 days. Children aged 6–11 months (or weighing 4.5–8 kg) received the infant dose (67.5 mg AQ/25 mg AS tablets) and children aged 12–59 months (or weighing 9–17 kg) received the child dose (136 mg AQ/50 mg AS tablets). Caregivers were responsible for administering the treatments to their child as instructed by the health worker.

### Randomization and blinding

A computer-generated randomization list (in blocks of 10) was created for each site by a member of the team who was not directly involved in patient recruitment, consultation or follow-up. Prior to study initiation, individual treatment allocation slips were prepared from the randomization list. These were sealed into sequentially-numbered, opaque envelopes containing the treatment group assignments. The consulting health worker opened the envelopes and assigned the treatment number and corresponding treatment at the time of prescription. Health facility nurses were responsible for dispensing the study medications according to the assigned study number. Study medications were not identical in appearance or taste nor were the tablets per dose the same. The participants, health workers, and study team were not blinded to the treatment assignments.

### Follow-up

Study participants were visited at home 4 days after their clinic visit; the day after the last treatment dose should have been taken. The purpose of the follow-up visit was explained to the caregivers, and additional written informed consent for the interview was obtained; participation was voluntary [27]. If the participating child required further medical attention at the time of the home visit, s/he was immediately referred to the nearest health centre, and the adherence assessment was conducted the following day, if the participant’s condition had improved. No interviews were carried out more than 5 days after the initial clinic visit.

A semi-structured questionnaire was administered to caregivers to assess adherence to treatment, the characteristics of the child and caregiver, knowledge of malaria, household characteristics, and wealth indicators. Caregivers were asked to describe the treatment, including how the medication was administered and any adverse events, and to show the original medication packaging. If the packaging was available, any remaining tablets were tallied and recorded onto the questionnaire. If treatment had not been completed at the time of the follow-up visit, the caregiver was encouraged to complete the full treatment course or, if that was not possible, to return to the health facility.

### Statistical methods

#### Sample size

In 2010, the prevalence of adherence to co-packaged AQ + AS in Sierra Leone was estimated to be 50% [20]. Based on studies in other countries, it was estimated that fixed-dose AQAS would yield a higher level of adherence (conservatively estimated to be 75%) [24, 28]. At the time of the study, there was no data on adherence levels to AL in Sierra Leone, however, based on the literature it was hypothesized that adherence to AL would be greater than co-packaged AQ + AS, but less than fixed-dose AQAS [3, 29]. In order to determine a 15% or greater absolute difference between the different treatment groups (two-sided test, 5% significance level, 80% power, 20% contingency (i.e. loss to follow-up, missing data etc.), a total of 198 patients were required for each treatment arm. As differences in context, health system and/or socioeconomic factors may influence adherence, the study was powered to detect differences in adherence to the two treatments, separately at each site. Thus, the sample size for each site was 400.

#### Outcome measures

Using previous ACT adherence studies as a guide, the primary outcome of caregiver adherence was based on self-reports of completion of treatment and, when possible, verified by package inspection [3, 29, 30]. Adherence was classified into four categories: (1) “definitely adherent”: caregiver reported completion of treatment and verified by an empty package; (2) “probably adherent”: reported completion of treatment but no package available for verification; (3) “probably non-adherent”: reported non-completion of treatment, but no package available for verification; and (4) “definitely non-adherent”: reported non-completion of the treatment verified by a package with remaining tablets.

As the main outcome variable was derived from two different measurements, the variable was recoded to produce two binary variables representing each component. These included: (1) “self-reported adherence”, which does not consider package availability (thus, self-reported adherence = definitely adherent + probably adherent; non-adherence = probably non-adherent + definitely non-adherent); and (2) “package-based adherence”, which does not consider self-reported adherence (thus package-based adherence = definitely adherent; non-adherence = probably adherent + probably non-adherent + definitely non-adherent).

Secondary outcomes were adherence to the prescribed number of doses, time-schedule and duration of treatment. The correct number of doses was defined as receiving the prescribed number of tablets, as indicated. Correct timing was defined as receiving the ACT at the prescribed intervals (twice daily for AL and once daily for AQAS). Correct duration was defined as receiving the ACT for the recommended number of days (3 days for both regimens). Correct treatment was defined as the composite of the three above indicators: correct dose + correct timing + correct duration, in which all three factors were met.

#### Data management and analysis

Data were recorded on paper forms and entered into a database created in Epi Info™ 7.1.2.0 (Centers for Disease Control and Prevention (CDC), Atlanta, GA USA). Data were double entered into tablets using the Epi Info Companion for Android mobile application. Statistical analysis was performed using Stata 12 (StataCorp, College Station, TX USA).

Although intention-to-treat analysis is the preferred analytic approach for randomized controlled trials, a per-protocol analysis was favoured for this trial as the primary outcome was adherence. The main objective of this study was to assess the behaviours of caregivers related to the specific ACT received at the health facility. Therefore, the primary analyses were carried out using the per-protocol population, in which only children who received ACT as per the randomization schedule and had outcome data were included. Children who did not receive the correct ACT regimen based on the randomization list were excluded from this analysis. We also conducted and present the intention-to-treat analysis for comparison with the per-protocol findings, to assess whether results from the two analytic approaches were similar. Participant’s characteristics and adverse events were tabulated by study site and randomization group in the per-protocol population. The wealth index was created using principle component analysis (PCA) [31].

Chi squared or Fisher’s exact tests were used to compare categorical data, and continuous data were tested using Student’s or Welch’s *t*-tests. Measures of effect (odds ratios-OR) were calculated using logistic regression for binary outcomes and ordinal logistic regression for multinomial outcomes along with the 95% confidence intervals and associated p-values. For the ordinal logistic regression model, the proportional odds assumption was tested with a likelihood ratio test.

### Ethical considerations

The study protocol was approved by the London School of Hygiene and Tropical Medicine (LSHTM) Research Ethics Committee and the Sierra Leone Ethics and Scientific Review Committee. The trial was registered at ClinicalTrials.gov (NCT01967472; https://clinicaltrials.gov/ct2/show/NCT01967472). All participants provided written informed consent at the time of recruitment and again prior to administration of the follow-up survey in their homes.

## Results

### Enrolment

**Fig. 2:**
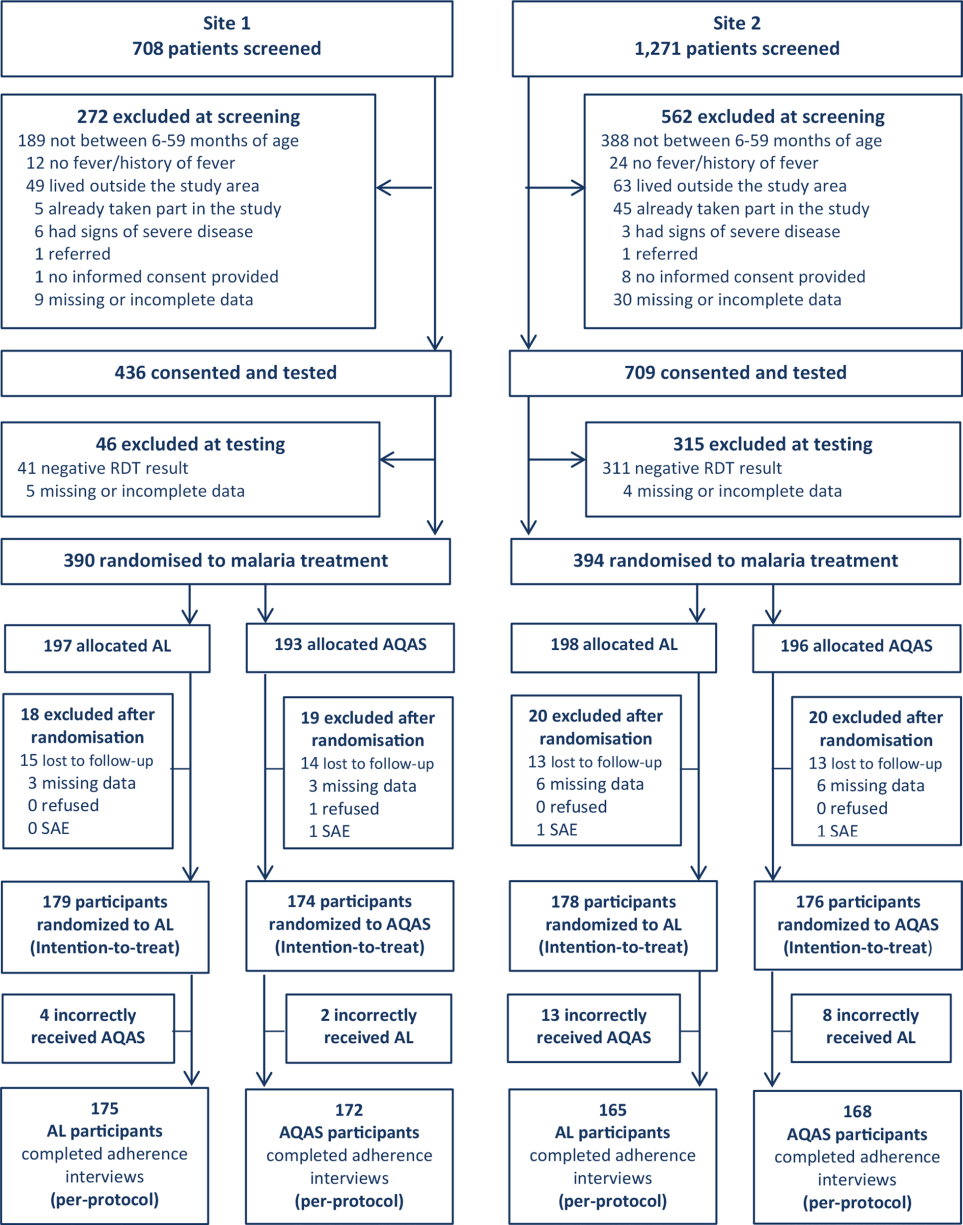
Study profile. *RDT*, rapid diagnostic test; *AQAS*, fixed-dose combination amodiaquine–artesunate; *AL*, artemether–lumefantrine; *SAE*, serious adverse event

The study was conducted from September 2013 to January 2014. Of the 1979 children screened (Fig. 2), 834 were excluded at screening, and 361 were excluded after testing negative for malaria. A total of 784 children were randomized to malaria treatment (390 at Site 1; 394 at Site 2); of these, 77 (9.7%) were excluded after randomization (lost to follow-up = 55, missing data = 18, refused 1, serious adverse event = 3; Fig. 2). The total number of children analysed in the intention-to-treat population was 707 (353 at Site 1 and 354 at Site 2). Treatment was misallocated in an additional 27 cases (6 at Site 1; 21 at Site 2); these children were excluded from the per-protocol analysis. Thus, 680 (85.6%) randomized children were included in the final per-protocol analysis (347 at Site 1; 333 at Site 2). Analyses were carried out for both the intention-to-treat and per-protocol populations, but all tables present the results from the per-protocol population. The intention-to-treat analysis is also presented for both the primary and secondary outcomes, but there is little if any difference between the findings.

### Characteristics of participants, caregivers, and households

The characteristics of participants and their caregivers and households were significantly different at the two study sites, therefore, all results are presented stratified by site (Table 1). Participants at Site 1 were younger than those at Site 2 (mean age 15 months vs 24 months, respectively, p < 0.001). No child at either site had been treated previously with AL, while some had received fixed-dose AQAS (41.3% at Site 1; 28.6% at Site 2; p = 0.025). Few caregivers reported that their child disliked AL, but complaints about AQAS were more common (19.8% at Site 1; 15.5% at Site 2; p = 0.550). At both sites, only caregivers in the AQAS arm reported that their child complained of bitter taste.

**Table 1:** Characteristics of participants, caregivers and households^a^

	Site 1		Site 2	
	AL (N = 175)	AQAS (N = 172)	AL (N = 165)	AQAS (N = 168)
*Participant characteristics*				
Weight^b^, kg [median (IQR)]	10 (8, 11)	10 (8, 11.4)	10 (8, 13)	10 (9, 13)
Age, months [median (IQR)]	15 (10, 24)	16 (11, 32.5)	24 (14, 37)	24 (14, 42.5)
Age categorized				
6 to 24 months	132 (75.4%)	116 (67.4%)	88 (53.3%)	85 (50.6%)
25 to 59 months	43 (24.6%)	56 (32.6%)	77 (45.7%)	83 (49.4%)
Gender (% female)	82 (46.9%)	72 (41.9%)	79 (47.9%)	76 (45.2%)
Previously taken the antimalarial treatment				
No	173 (98.9%)	97 (56.4%)	165 (100%)	118 (70.2%)
Yes	0 (0.0%)	71 (41.3%)	0 (0.0%)	48 (28.6%)
Unknown	2 (1.1%)	4 (2.3%)	0 (0.0%)	2 (1.2%)
Disliked the antimalarial treatment				
No	156 (89.1%)	128 (74.4%)	153 (92.7%)	130 (77.4%)
Yes	2 (1.1%)	34 (19.8%)	7 (4.2%)	26 (15.5%)
Unknown	17 (9.7%)	10 (5.8%)	5 (3.0%)	12 (7.1%)
Complained of bitter taste	0 (0.0%)	31 (18.0%)	0 (0.0%)	18 (10.7%)
*Caregiver characteristics*				
Age, years [median (IQR)]	25 (21, 30)	25 (21, 29)	27 (22, 34)	27 (23, 34)
Gender (% female)	169 (96.6%)	166 (96.5%)	156 (94.6%)	162 (96.4%)
Fluent in Krio	123 (70.3%)	124 (72.1%)	116 (70.3%)	118 (70.2%)
Any education	107 (61.1%)	86 (50.0%)	109 (66.1%)	116 (69.1%)
Told to finish treatment by health worker	101 (57.7%)	101 (58.7%)	85 (51.5%)	73 (43.5%)
Knowledge about ACTs	31 (17.7%)	35 (20.4%)	54 (32.7%)	51 (30.4%)
*Household characteristics*				
Religion^c^				
Christian	27 (15.4%)	22 (12.8%)	77 (46.7%)	91(54.2%)
Muslim	148 (84.6%)	150 (87.2%)	87 (52.7%)	77 (45.8%)
Household wealth index^d^				
1 (poorest)	40 (23.7%)	47 (27.5%)	77 (47.0%)	72 (43.1%)
2	59 (34.9%)	61 (35.7%)	42 (25.6%)	47 (28.1%)
3 (least poor)	70 (41.4%)	63 (36.8%)	45 (27.4%)	48 (28.7%)

^a^Demographic data were collected during follow-up visits; therefore, this information is only available from participants that were located and consented for the follow-up interviews (the per-protocol population)

^b^Scale availability was problematic at both sites on select days, so weight data is not available for some participants. Denominators for weight: Site 1: AL = 165; AQAS-164; Site 2: AL = 151; AQAS = 152

^c^Religion: information on religion is missing from one participant in the AL group at Site 2 (n = 164)

^**d**^Wealth index denominators: Site 1 AL = 169 and AQAS = 171; Site 2 AL = 164 and AQAS = 167

Caregivers were also younger at Site 1 (median age of 25 years at Site 1 vs 27 years at Site 2, p < 0.001); most caregivers (> 70%) spoke Krio (the local language) at both sites. In Site 1, caregivers in the AL arm were more educated than those in the AQAS arm (61.1% vs 50.0%, respectively, p = 0.040). At both sites, approximately half of caregivers reported that health workers instructed them to finish the anti-malarial treatment. Caregiver knowledge about ACT was low at both sites, more so in Site 1.

The Muslim religion was practised by substantially more households at Site 1 than at Site 2 (85.9% vs 49.2%, respectively, p < 0.001). Households at Site 2 were significantly poorer than at Site 1, with 149 (44.7%) households assigned to the poorest category at Site 2 vs 87 (25.1%) in Site 1 (p < 0.001). Otherwise, there were no additional differences in characteristics of participants, caregivers or households, between trial arms, at either site.

### Adherence

At both sites, the odds of definite adherence (defined as self-reported adherence in the presence of an empty drug package) were higher for AL than AQAS (Table 2). Self-reported adherence (ignoring the results of the drug package inspection) was > 90% for both regimens, but varied between the sites. At Site 1, self-reported adherence to AL was slightly higher than to AQAS, but at Site 2, self-reported adherence to AQAS was greater than to AL. However, adherence determined by inspecting drug packaging alone was higher for AL than AQAS at both sites; adherence (defined by empty packaging) to both regimens was higher at Site 1 than Site 2. This variability in findings reflects differences in the retention of the drug package by caregivers, which was significantly higher at Site 1 than at Site 2 (263 [75.8%] vs 185 [52.6%], respectively, p < 0.001; Additional file 1). In addition, at both sites, significantly more caregivers saved AL packages than AQAS packages (Site 1: AL 147 [84.0%] vs AQAS 116 [67.4%], p < 0.001; Site 2: AL 103 [62.4%] vs AQAS 72 [42.9%], p < 0.001).

**Table 2:** Primary adherence outcomes

	Site 1			Site 2		
	AL	AQAS	OR (95%CI) p-value*	AL	AQAS	OR (95%CI) p-value*
Per-Protocol	N = 175	N = 172		N = 165	N = 168	
Primary adherence outcome^a^						
Definitely non-adherent	2 (1.1%)	3 (1.7%)	2.16 (1.34− 3.49) 0.001	10 (6.1%)	5 (3.0%)	1.53 (1.00− 2.33) 0.049
Probably non-adherent	4 (2.3%)	4 (2.3%)		4 (2.4%)	1 (0.6%)	
Probably adherent	30 (17.1%)	56 (32.6%)		65 (39.4%)	99 (58.9%)	
Definitely adherent	139 (79.4%)	109 (63.4%)		86 (52.1%)	63 (37.5%)	
Self-reported adherence^b^						
Non-adherent	6 (3.4%)	7 (4.1%)	1.19 (0.39− 3.63) 0.753	14 (8.5%)	6 (3.6%)	0.40 (0.15− 1.07) 0.067
Adherent	169 (96.6%)	165 (95.9%)		151 (91.5%)	162 (96.4%)	
Adherence based on packaging^c^						
Non-adherent	36 (20.6%)	63 (36.6%)	2.23 (1.38− 3.61) 0.001	79 (47.9%)	105 (62.5%)	1.81 (1.17− 2.81) 0.008
Adherent	139 (79.4%)	109 (63.4%)		86 (52.1%)	63 (37.5%)	

Intention-to-treat	N = 179	N = 174		N = 178	N = 176	
Primary adherence outcome^d^						
Definitely non-adherent	2 (1.1%)	3 (1.7%)	2.15 (1.35− 3.44) 0.001	11 (6.2%)	6 (3.4%)	1.40 (0.93− 2.10) 0.109
Probably non-adherent	4 (2.3%)	4 (2.3%)		4 (2.3%)	1 (0.6%)	
Probably adherent	32 (17.9%)	58 (33.3%)		73 (41.0%)	101 (57.4%)	
Definitely adherent	141 (78.8%)	109 (62.6%)		90 (50.6%)	68 (38.6%)	
Self-reported adherence^b^						
Non-adherent	6 (3.4%)	7 (4.0%)	1.21 (0.40− 3.67) 0.738	15 (8.4%)	7 (4.0%)	0.45 (0.18− 1.13) 0.090
Adherent	173 (96.7%)	167 (96.0%)		163 (91.6%)	169 (96.0%)	
Adherence based on packaging^c^						
Non-adherent	38 (21.2%)	65 (37.4%)	2.21 (1.38− 3.55) 0.001	88 (49.4%)	108 (61.4%)	1.62 (1.06− 2.48) 0.024
Adherent	141 (78.8%)	109 (62.6%)		90 (50.6%)	68 (38.6%)	

*Odds Ratios were calculated using ordinal logistic regression for the primary adherence outcome and logistic regression for the binary outcomes with ORs calculated using AQAS as the reference group

^a^Definitely adherent = self-reported adherence + empty package; probably adherent = self-reported adherence (no package); probably non-adherent = self-reported non-adherence (no package); definitely non-adherent = self-reported non-adherence + package with tablets remaining

^b^Adherent = definitely adherent + probably adherent

^c^Adherent = only definitely adherent

### Quality of treatment

Overall, the quality of treatment was high (Table 3). At both sites, nearly all participants received the total required tablets for both regimens. At Site 1, the mean proportion of number of tablets taken was higher, but not significantly, for AL (99.8%), while the opposite was found at Site 2, where the mean proportion of tablets received was marginally higher for AQAS (99.2%). Subtle differences in treatment patterns were found at both sites. At Site 1, significantly fewer AQAS participants received the appropriate number of tablets for their weight (correct dose), but fewer AL participants were treated the correct number of times per day (correct timing), and for the correct number of days (correct duration), although these findings were not statistically significant. At Site 2, AL participants were significantly less likely to be treated with the appropriate number of tables and for the correct number of times per day (correct dose and correct timing), and were less likely to receive the correct treatment overall (correct dose + timing + duration), than AQAS participants.

**Table 3:** Quality of treatment with ACT

Per-Protocol	Site 1					Site 2				
Treatment adherence^a^	AL (N = 176)	AQAS (N = 176)	Difference in means	95% CI	p-value	AL (N = 173)	AQAS (N = 181)	Difference in means	95% CI	p-value
Mean % of treatment (SD)^b^	99.8% (2.5)	98.6% (7.9)	1.17	− 0.09, 2.42	0.068	97.0% (12.8)	99.2% (6.3)	− 2.19	− 4.37, 0.00	0.050

Correct treatment^c^	AL (N = 179)	AQAS (N = 174)	Odds Ratio	95% CI	p-value	AL (N = 178)	AQAS (N = 176)	Odds ratio	95% CI	p-value
Correct dose^d^	163 (93.1%)	144 (83.7%)	2.64	1.30, 5.39	0.008	128 (77.6%)	149 (88.7%)	0.44	0.24, 0.81	0.008
Correct timing^e^	163 (93.1%)	166 (96.5%)	0.49	0.18, 1.34	0.165	136 (82.4%)	163 (97.0%)	0.14	0.05, 0.38	< 0.001
Correct duration^f^	172 (98.3%)	170 (98.8%)	0.67	0.11, 4.09	0.668	156 (95.6%)	164 (97.6%)	0.42	0.13, 1.40	0.159
Correct treatment^g^	154 (88.0%)	140 (81.4%)	1.68	0.92, 3.04	0.089	125 (75.8%)	148 (88.1%)	0.42	0.23, 0.76	0.004

Intention-to-treat	Site 1					Site 2				
Treatment adherence^a^	AL (N = 175)	AQAS (N = 172)	Difference in means	95% CI	p-value	AL (N = 165)	AQAS (N = 168)	Difference in means	95% CI	p-value
Mean % of treatment (SD)^b^	99.8% (2.5)	98.7% (7.9)	1.14	− 0.09, 2.36	0.069	97.0% (12.7)	99.1% (6.0)	− 2.30	− 4.40, − 0.19	0.032

Correct treatment^c^	AL (N = 175)	AQAS (N = 172)	Odds Ratio	95% CI	p-value	AL (N = 165)	AQAS (N = 168)	Odds Ratio	95% CI	p-value
Correct dose^d^	166 (92.7%)	146 (83.9%)	2.45	1.22, 4.90	0.011	138 (77.5%)	155 (88.1%)	0.47	0.26, 0.83	0.010
Correct timing^e^	166 (92.7%)	168 (96.6%)	0.45	0.17, 1.23	0.120	149 (83.7%)	170 (96.6%)	0.18	0.07, 0.45	< 0.001
Correct duration^f^	179 (97.8%)	174 (98.9%)	0.51	0.09, 2.81	0.439	169 (94.9%)	171 (97.2%)	0.55	0.18, 1.67	0.291
Correct treatment^g^	156 (87.2%)	142 (81.6%)	1.53	0.85, 2.74	0.153	135 (75.8%)	154 (87.5%)	0.45	0.26, 0.79	0.005

^a^Proportion of all tablets received − information on the number of tablets taken is missing from one participant in the AL group at Site 1 (n = 174). Measure of Effect = Difference of means; AQAS is the reference group

^b^Percent of treatment = total number of tablets taken by the patient/total number of tablets prescribed

^c^Odds Ratios were calculated using logistic regression, with ORs calculated using AQAS as the reference group

^d^Dose is defined as the number of tablets prescribed by weight or age. For AQAS: 1 tablet per day for three days. For AL: 5 to < 15 kg = 1 tablet twice a day for 3 days, 15 to < 25 kg = 2 tablets twice a day for three days

^e^Timing is defined as the number of times per day the treatment should be taken. For AQAS: once daily. For AL: two times per day

^f^Duration is defined as the number of days the treatment should be taken. For both AQAS and AL: 3 days

^g^Correct treatment = correct dose + correct timing + correct duration, in which the criteria for all three factors (as above) are met

### Adverse events

A total of 106 caregivers reported that their child experienced an adverse event to treatment. Significantly more adverse events were reported at Site 2 than at Site 1 (66 [19.8%] vs 33 [9.5%], respectively, p < 0.001). At both sites, significantly more caregivers in the AQAS arm reported adverse events (Table 4), with vomiting, weakness-fatigue, and dizziness most commonly reported. At Site 1, significantly more caregivers in the AQAS arm reported that their child vomited, or was weak or dizzy. Similar results were seen at Site 2, but only weakness was reported by significantly more caregivers in the AQAS arm. Three serious adverse events, including two hospitalizations for severe malaria and one death, were reported during the study period, but none were felt to be related to the study medications. The cause of death was unknown for the child that died. However, the child was noted by the study staff to have danger signs suggestive of severe disease, and was referred, but unfortunately the caregiver did not seek further care.

**Table 4:** Reported adverse events (side effects)

	Site 1			Site 2		
	AL (N = 175)	AQAS (N = 172)	p-value*	AL (N = 165)	AQAS (N = 168)	p-value*
Any adverse event						
Caregiver reported	6 (3.4%)	27 (15.7%)	< 0.001	25 (15.2%)	41 (24.4%)	0.039
Specific adverse events						
Vomiting	4 (2.3%)	16 (9.3%)	0.005	13 (7.9%)	18 (10.7%)	0.452
Weakness-Fatigue	0 (0.0%)	12 (7.0%)	< 0.001	9 (5.5%)	29 (17.3%)	0.001
Dizziness	0 (0.0%)	10 (5.8%)	0.001	7 (4.2%)	4 (2.4%)	0.376
Diarrhoea	1 (0.6%)	3 (1.7%)	0.369	2 (1.2%)	2 (1.2%)	1.000
Other gastrointestinal complaints^a^	1 (0.6%)	5 (2.9%)	0.119	2 (1.2%)	4 (2.4%)	0.685
Other AE reported^b^	1 (0.6%)	2 (1.2%)	0.621	4 (2.4%)	4 (2.4%)	1.000

* Fisher’s exact test

^a^Reported one or more of the following: diarrhoea, anorexia, nausea or abdominal pain

^b^ Other AE’s reported: Site 1— AL: 1 pruritic; AQAS: 1 headache & 1 not specified. Site 2—AL: 2 change in urine colour, 1 cold/flu, & 1 sweating; AQAS: 1 change in urine colour, 1 fever, 1 mouth sores & 1 unspecified

## Discussion

With progress on malaria control slowing and resistance to artemisinin resistance emerging, it is vital that every effort is made to protect the efficacy of ACT [32]. Patient adherence to prescribed anti-malarial regimens is a key step in the pathway to treatment effectiveness [4]. Yet, evidence on the impact of co-formulation of drug regimens, and comparative data on adherence to available ACT, is limited [3]. In this randomized trial, self-reported adherence was high for both co-formulated AL and AQAS, but varied by study site. At both sites, definite adherence was significantly higher for AL than AQAS. However, this outcome was influenced by the likelihood of retention of the drug package by caregivers, which was significantly higher for AL. Overall, the quality of treatment was high; however, disadvantages to both regimens were identified that could negatively impact adherence. AL was less likely to be administered correctly at one site, which is not surprising given the greater complexity of the dosing regimen. AQAS was less well-tolerated at both sites, and was associated with bitter taste and significantly more adverse events. To better understand adherence to ACT, and how adherence might impact on treatment effectiveness, additional studies are warranted, particularly comparative studies including ACT and new drug regimens as these become available. Standardizing methodologies for evaluating adherence would also improve the evidence base on ACT adherence.

Currently, the available evidence on ACT adherence is limited by variation in study designs and outcomes, differences in drug regimens, and lack of comparative studies [3]. In prior randomized trials, adherence to AL has ranged from 64 to 99% [33–38]. Similarly, adherence to co-packaged AQ + AS and AQAS has varied widely in prior studies. In Sierra Leone, adherence to co-packaged AQ + AS was only 48% [20], while in Zanzibar and Ghana adherence to AQ + AS was much higher (77 and 93%, respectively) [39, 40]. Two recent studies from The Democratic Republic of Congo reported adherence to fixed-dose AQAS to be 75 and 62% [41, 42], and in a study in Madagascar, adherence to AQAS was even higher (90%) [28].

The one other study that directly compared adherence to AL and AQAS in Benin found that ‘full adherence’ to the two regimens was not significantly different [24]. However, this study primarily evaluated treatment effectiveness, with adherence as a secondary outcome; little information was provided about how adherence was defined and measured. Apparently, adherence was assessed during a home visit on Day 3 of treatment and drug packages were collected when available, but it is not clear how ‘full adherence’ was defined, limiting the ability to compare their results to findings from this study. The results reported here build on the available evidence, suggesting that self-reported adherence to both AL and AQAS are high, and highlighting methodological challenges that should be addressed in future studies.

This study also identified specific characteristics which may impact adherence to AL and AQAS. AL was less likely to be taken correctly at one site, but was better tolerated than AQAS at both sites. The complexity of the AL dosing regimen, including the number of tablets, twice daily dosing, the requirement to give the second dose 8 h after the first, and to administer with fatty food [43], has been shown to negatively impact treatment adherence [44, 45]. In contrast, while AQAS has been optimized to be dosed only once daily [46–48], its bitter taste and greater likelihood of adverse events make it more difficult to administer to children [49, 50], and may reduce adherence as found in this study. Pharmaceutical companies have focused on producing child-friendly ACT formulations, including smaller tablets, dispersible tablets, and improved weight-for-age dosing recommendations [10, 12, 51, 52]. As new anti-malarial drugs are developed and evaluated for effectiveness, it will be important to assess child-friendly regimens that are palatable and easy to administer, in order maximize treatment adherence and outcomes in those affected most by malaria [53].

In this study, adherence to AL and AQAS varied depending on the outcome definition applied. Although definitions of adherence outcomes, based on self-report of treatment completion plus package inspection, which have been used in previous anti-malarial studies were adopted [3, 29, 30], limitations to these definitions were found. Both indicators used to define adherence are subject to bias. Self-reported adherence is open to social desirability bias, with caregivers more likely to report what they perceive to be ‘correct’ answers, potentially leading to over-estimation of adherence. Package inspection and pill counts, while more ‘objective’ measures [54, 55], are dependent on the availability of packaging. In the Benin study, more AQAS packages were found (84.4% of AL vs 93.7% of AQAS packages) [24], in contrast to this study, in which more AL packages were retained. Factors that influence desirability of retaining packaging could also impact on adherence. Prior research suggests that novel drug packages, that aim to educate or market a drug regimen, may be more attractive or appealing, and thus may be more likely to be retained [56, 57], thus impacting on measures of adherence that incorporate data from package examinations, or may impact adherence directly [58].

A variety of approaches have been used to incorporate package inspection in the classification of adherence outcome [36, 59, 60]. Under trial conditions, blister packages have been retained and inspected with results successfully incorporated into the outcome measurement [61]. In other studies, package inspection has been included in the methods, but either excluded from the analysis [62], or not utilized altogether. For example, although the packaging was part of the outcome definition, the proportion of packaging available was not reported in a number of studies [35, 41, 42]. In others, the package information although collected, was not utilized and only self-reported (probable) adherence rates were reported [44, 63, 64]. In Ghana, an intervention study used package inspection as a secondary outcome to validate self-reported adherence, but found that only 60% of patients were able to produce their package, suggesting that this may not be the most accurate measurement of ACT adherence [36]. Likewise, in Ethiopia, difficulties with package retention over many days were reported, suggesting that presence of packaging may not be indicative of true adherence [50].

Although package inspections and pill counts serve as a gold standard for measuring adherence to treatment of other diseases [54, 65] the heterogeneity of this outcome measure in malaria studies limits comparison of adherence to regimens across studies. Instead, examining the quality of treatment, including whether the correct number of tablets were given at the correct frequency for the correct number of days, may provide a more accurate picture of treatment adherence [35, 37, 59, 63, 66]. Recently studies measuring anti-malarial adherence have presented per-dose adherence measurements [36, 59, 61], this approach is useful as it can illustrate at which point patients stop taking their medications. However, this approach like those mentioned earlier relies on self-report, with or without package validation. Standardizing methodologies for evaluating adherence is necessary to improve the evidence base on ACT adherence.

This study had several limitations, in addition to the challenges with the adherence outcome classification. First, the characteristics of the participants, caregivers, and households enrolled in the two sites varied substantially, which was unexpected. Specifically, variations in the age of participating children, level of caregiver education, household religion and socioeconomic position were found, all of which may influence treatment adherence [3, 29, 67, 68]. To address these differences, the analysis was stratified by study site. Stratification did not impact on the power to detect differences in adherence outcomes, as the sample size calculations were done independently for the two sites, in case differences in the sites were found. Second, an unexpectedly high number of children were excluded after testing with RDT and after randomization, particularly at Site 2. In addition, several children received the incorrect treatment, again more commonly at Site 2. These exclusions and the imbalance between sites were likely due to characteristics of the health centres, and random error. No systematic biases were suspected. Furthermore, to examine the validity of the findings, a sensitivity analysis was conducted comparing outcome measures in the ITT and PP populations, which found that outcome measurements were almost identical for both analytical approaches, suggesting that the exclusions after randomization did not impact the outcomes presented here. Third, the design of this study may have led to an overestimation of adherence. Effectiveness trials and cross-sectional studies have been shown to report higher adherence levels than prospective observational studies; however, even results from such studies vary [3, 30]. Moreover, although this study did occur under ‘normal conditions’, the fact that providers and caregivers were observed, may have altered their behaviour as a result of participating in the study (participation bias), and influenced adherence outcomes [29, 69]. Finally, the limitations of only looking at statistical significance should be noted, as statistically significant differences do not always equate to clinical importance. This study was powered to detect a 15% difference in rates of adherence between AL and AQAS, with the thinking that if the absolute difference in adherence between the two regimens was 15% or more that this would a big enough difference from a clinical or public health perspective to favour one treatment over the other. From a public health perspective, the findings of this study did not find a difference large enough to favour one regimen over another; however, the secondary outcome (correct treatment and its associated components) does hightlight operational areas where ACT administration could be improved.

## Conclusion

Maximizing adherence to anti-malarial drug regimens is essential for ensuring treatment effectiveness; however measuring adherence remains challenging. The results from this study suggest that although self-reported adherence to both AL and AQAS was high, the difference between the two regimens was not significant. However, potential disadvantages were identified for each regimen that might impact optimal treatment adherence. With the emergence of resistance to artemisinins in Southeast Asia fuelling the development of new drug formulations, information on adherence to different ACT regimens will become increasingly important to help guide drug delivery, improve treatment effectiveness, and inform drug policy. However, the methodology of measuring adherence in anti-malarial studies requires further advancement. This study highlights the limitations of package inspection, and suggests that an outcome measure based on correct treatment could have greater utility. Standardizing methodologies for evaluating adherence across diverse contexts would improve the evidence base on ACT adherence and effectiveness.

## Additional file

**Additional file 1:Table S1.** Package availability by drug and site

## Abbreviations

ACT: artemisinin-based combination therapy
AL: Artemether–lumefantrine
AQAS: fixed-dose combination amodiaquine–artesunate
AQ + AS: co-packaged amodiaquine plus artesunate
DRC: The Democratic Republic of Congo
FDC: fixed-dose combination
GFATM: The Global Fund to Fight AIDS, Tuberculosis and Malaria
ITT: intention-to-treat
MDA: mass drug administration
NMCP: National Malaria Control Programme
PP: per-protocol
RDT: rapid diagnostic test
WHO: World Health Organization
95% CI: 95% confidence interval
