# Supplementary material for: Adherence to treatment with artemether–lumefantrine or amodiaquine–artesunate for uncomplicated malaria in children in Sierra Leone: a randomized trial
Source: Malar J. 2018 Jun 4;17:222. doi: 10.1186/s12936-018-2370-x (PMC5987409; doi:10.1186/s12936-018-2370-x)
Supplement: Supplementary file 1 — Additional file 1:Table S1. Package availability by drug and site [file 12936_2018_2370_MOESM1_ESM.docx]

**Additional File 1. Package Availability**

**Table S1. Package availability**

|  | **By Site** | | |  | **By Drug** | | |  |
| --- | --- | --- | --- | --- | --- | --- | --- | --- |
|  | **Site 1** | **Site 2** |  |  | **AL** | **AQAS** |  |  |
| **Intention to treat analysis** | **N=353** | **N=354** | **P value*** |  | **N=350** | **N=357** | **P value*** |  |
| **Package available** |  |  |  |  |  |  |  |  |
| —No | 87 (24.7%) | 169 (47.7%) | **<0.001** |  | 94 (26.9%) | 162 (45.4%) | **<0.001** |  |
| —Yes | 266 (75.4%) | 185 (52.3%) |  |  | 256 (73.1%) | 195 (54.6%) |  |  |
| **Per protocol analysis** | **N=347** | **N=333** | **P value*** |  | **N=340** | **N=340** | **P value*** |  |
| **Package available** |  |  |  |  |  |  |  |  |
| —No | 84 (24.2%) | 158 (47.5%) | **<0.001** |  | 90 (26.5%) | 152 (44.7%) | **<0.001** |  |
| —Yes | 263 (75.8%) | 175 (52.6%) |  |  | 250 (73.5%) | 188 (55.3%) |  |  |

*chi-squared test

**Table S2. Package availability by site and drug**

|  | **Site 1** | | |  | **Site 2** | | |  |
| --- | --- | --- | --- | --- | --- | --- | --- | --- |
|  | **AL** | **AQAS** |  |  | **AL** | **AQAS** |  |  |
| **Intention to treat analysis** | **N=179** | **N=174** | **P value*** |  | **N=178** | **N=176** | **P value*** |  |
| **Package available^1^** |  |  |  |  |  |  |  |  |
| —No | 29 (16.2%) | 58 (33.3%) | **<0.001** |  | 71 (39.9%) | 98 (55.7%) | **0.003** |  |
| —Yes | 150 (83.8%) | 116 (66.7%) |  |  | 107 (60.1%) | 78 (44.3%) |  |  |
| **Per protocol analysis** | **N=175** | **N=172** | **P value*** |  | **N=165** | **N=168** | **P value*** |  |
| **Package available** |  |  |  |  |  |  |  |  |
| —No | 28 (16.0%) | 56 (32.6%) | **<0.001** |  | 62 (37.6%) | 96 (57.1%) | **<0.001** |  |
| —Yes | 147 (84.0%) | 116 (67.4%) |  |  | 103 (62.4%) | 72 (42.9%) |  |  |

*chi-squared test
